# Utility of NT-proBNP as an objective marker of postoperative heart failure after coronary artery bypass surgery: a prospective observational study

**DOI:** 10.1186/s13741-021-00194-4

**Published:** 2021-07-13

**Authors:** Huiqi Jiang, Jonas Holm, Örjan Friberg, Farkas Vanky, Mårten Vidlund, Bashir Tajik, Yanqi Yang, Rolf Svedjeholm

**Affiliations:** 1 Department of Cardiothoracic Surgery and Anesthesia, Faculty of Medicine and Health Sciences, Department of Health, Medicine and Caring Sciences, Linköping University, SE-581 85 Linköping, Sweden; 2 Department of Cardiothoracic Surgery, Sun Yat-Sen Memorial Hospital, Sun Yat-sen University of Medical Sciences, Guangzhou, Guangdong China; 3 Department of Cardiothoracic and Vascular Surgery, Faculty of Medicine and Health, Örebro University, Örebro, Sweden

**Keywords:** Natriuretic peptide, Heart failure, Postoperative care, Coronary artery bypass surgery

## Abstract

**Background:**

Postoperative heart failure (PHF) is the main cause for mortality after cardiac surgery but unbiased evaluation of PHF is difficult. We investigated the utility of postoperative NT-proBNP as an objective marker of PHF after coronary artery bypass surgery (CABG).

**Methods:**

Prospective study on 382 patients undergoing isolated CABG for acute coronary syndrome. NT-proBNP was measured preoperatively, the first (POD1) and third postoperative morning (POD3). A blinded Endpoints Committee used prespecified criteria for PHF. Use of circulatory support was scrutinized.

**Results:**

After adjusting for confounders PHF was associated with 1.46 times higher NT-proBNP on POD1 (*p* = 0.002), 1.54 times higher on POD3 (*p* < 0.0001). In severe PHF, NT-proBNP was 2.18 times higher on POD1 (*p* = 0.001) and 1.81 times higher on POD3 (*p* = 0.019). Postoperative change of NT-proBNP was independently associated with PHF (OR 5.12, 95% CI 1.86–14.10, *p* = 0.002). The use of inotropes and ICU resources increased with incremental quartiles of postoperative NT-proBNP.

**Conclusions:**

Postoperative NT-proBNP can serve as an objective marker of the severity of postoperative myocardial dysfunction. Due to overlap in individuals, NT-proBNP is useful mainly for comparisons at cohort level. As such, it provides a tool for study purposes when an unbiased assessment of prevention or treatment of PHF is desirable.

**Trial registration:**

ClinicalTrials.gov Identifier: NCT00489827https://clinicaltrials.gov/ct2/show/NCT00489827?term=glutamics&draw=2&rank=1.

**Supplementary Information:**

The online version contains supplementary material available at 10.1186/s13741-021-00194-4.

## Background

Postoperative heart failure (PHF) accounts for the majority of deaths after cardiac surgery (O'Connor et al., 1998; Surgenor et al., 2001; Vanky et al., 2004). The Northern New England Cardiovascular Study group found that differences in postoperative mortality after coronary artery bypass surgery (CABG) were mainly explained by differences in mortality rates caused by PHF (O'Connor et al., 1998). The lack of universally accepted criteria for the diagnosis of PHF renders scientific evaluation of prevention and treatment of this important complication difficult (Gillies et al., 2005; Fellahi et al., 2008).

Some degree of myocardial dysfunction is seen in virtually every patient, even after routine coronary artery bypass surgery, despite modern techniques for myocardial protection (Breisblatt et al., 1990). Postoperative myocardial dysfunction usually is mild and transient and the threshold when inotropic treatment should be instituted varies markedly between different institutions and physicians (Fellahi et al., 2008; Mehta et al., 2017).

Defining heart failure is difficult under any circumstance as was illustrated by a survey among reviewers of Cardiovascular Research (Coronel et al., 2001). In cardiac surgery, it may seem straightforward to rely on cardiac output measurements for the definition as PHF is commonly termed low cardiac output syndrome. However, PHF usually presents at weaning from cardiopulmonary bypass or early after surgery when patients have a low systemic oxygen demand due to anesthesia and, hence, cardiac output can be very low even in patients with completely a normal postoperative course (Vanhanen et al., 1998; Hakanson et al., 1995). Mixed venous oxygen saturation (SvO_2_) reflects the balance between oxygen delivery to the tissues and systemic oxygen demand. Although there are pitfalls, such as shivering, anemia and hypovolemia, SvO_2_ in the early postoperative course is well documented with regard to outcome and furthermore the pitfalls are usually easily recognizable (Holm et al., 2011; Holm et al., 2010; Svedjeholm et al., 1999).

In the GLUTAmate for Metabolic Intervention in Coronary Surgery (GLUTAMICS) trial (ClinicalTrials.gov Identifier: NCT00489827), we used a blinded Clinical endpoints committee relying mainly on prespecified SvO_2_ criteria to diagnose PHF (Vidlund et al., 2012). The committee found the criteria easy to use but the meetings consumed a lot of time and resources. Furthermore, the study required pulmonary artery catheters in all patients. Alternative objective measures to facilitate assessment of PHF are therefore desirable.

In cardiology practice, natriuretic peptides have been established as biomarkers for diagnosis of heart failure and for evaluation of treatment (Ponikowski et al., 2016; Yancy et al., 2017).

In cardiac surgery, preoperative natriuretic peptides are recognized as predictors of postoperative morbidity, hospital mortality, and long-term survival (Fox et al., 2008; Young et al., 2014; Holm et al., 2014; Kerbaul et al., 2004; Mitchell & Webb, 2011; Lurati Buse et al., 2011). Preoperative risk assessment has overshadowed any role for postoperative natriuretic peptides, although high postoperative levels have been reported to be associated with adverse outcome, need for inotropic and mechanical circulatory support, and 1-year mortality (Fox et al., 2008; Young et al., 2014; Kerbaul et al., 2004; Reyes et al., 2005; Nozohoor et al., 2009; Crescenzi et al., 2009; Suttner et al., 2008; Provenchere et al., 2006; Mauermann et al., 2017). Only half a dozen studies have evaluated postoperative natriuretic peptides with regard to what could be considered PHF (Fox et al., 2008; Kerbaul et al., 2004; Reyes et al., 2005; Nozohoor et al., 2009; Suttner et al., 2008; Provenchere et al., 2006). None of the studies adjusted for variables not directly related to cardiac function known to influence plasma levels of NT-proBNP, such as age, gender, renal function, and obesity (Redfield et al., 2002; Wang et al., 2004; Chenevier-Gobeaux et al., 2005). Just two of these studies included more than hundred patients and both used treatment criteria for PHF (Fox et al., 2008; Nozohoor et al., 2009). Three studies used prespecified criteria for PHF but they were small and PHF accounted only for a proportion of postoperative complications that constituted the endpoint (Kerbaul et al., 2004; Suttner et al., 2008; Provenchere et al., 2006).

All in all available studies suggest an association between postoperative levels of natriuretic peptides and PHF. However, available data is limited, does not adjust for important non-cardiac variables, and these studies have not evaluated if natriuretic peptides reflect the severity of PHF. The aim of our study was to address these gaps in knowledge regarding the utility of postoperative NT-proBNP as an objective marker of PHF and its severity after CABG.

## Methods

### Ethical approval

Ethical approval for this substudy to the GLUTAMICS trial (original protocol no M76-05; addendum 26-07) was provided by the Regional Ethical Review Board in Linköping, Sweden. It was performed according to the Helsinki Declaration of Human Rights and the patients were enrolled in the study after written informed consent.

### Patients

The study was based on a previous clinical trial (GLUTAMICS) and done as preparation for its sequel GLUTAMICSII. The study population consisted of 382 patients with acute coronary syndrome undergoing urgent isolated first-time CABG who were enrolled in the GLUTAMICS-trial at three Swedish Cardiac Surgery centers (Linköping University Hospital, Örebro University Hospital, and Karlskrona Hospital) from May 30, 2007 to November 12, 2009 (Vidlund et al., 2012). The GLUTAMICS-trial was an investigator-initiated, prospective, randomized, placebo-controlled, double-blind trial with parallel assignment to glutamate or placebo (saline) (Vidlund et al., 2012). Detailed inclusion and exclusion criteria are presented in the Supplemental methods. Sample size was based on the GLUTAMICS-trial, which was powered with regard to the intervention and primary endpoint. The study was terminated per protocol after interim analysis because of prespecified stopping criteria (Vidlund et al., 2012). A flow chart of the patients in the substudy is given in Supplemental Figure S1. For further details, see the Supplemental methods.

### Study design

This was a prospective, observational, longitudinal cohort study to evaluate the association between postoperative NT-proBNP and prespecified criteria for PHF used in the GLUTAMICS-trial. A blinded Clinical Endpoints Committee decided if PHF or severe PHF had occurred (Vidlund et al., 2012). The committee consisted of consultants in cardiothoracic anesthesia and cardiothoracic surgery from the participating centers. They based their decisions on prespecified criteria mainly relying on mixed venous oxygen saturation (SvO_2_) supported by data from the case record forms, database, and hospital records (including echocardiographic examination, hemodynamic data, and use of circulatory support). The endpoints committee was blinded to the results of NT-proBNP analyses (Holm et al., 2014). The NT-proBNP results were released from the laboratory when the trial was terminated. External monitoring of all key study data was done by an independent professional monitoring team.

A retrospective analysis was added to study the association between different levels (quartiles) of postoperative NT-proBNP and the need for inotropic treatment. Use of inotropic drugs was detected by the aid of the Case Report Form (CRF), the institutional database and ICU-records. Use of inotropes in the OR and on admission to ICU was registered in the CRF.

Use of inotropes in the ICU was detected from the institutional database and ICU records. An investigator previously not involved in the GLUTAMIC-trial, scrutinized all ICU records with regard hourly registration of inotropic drug infusion, dosage, and total duration of treatment. Complete ICU-records were available for 171 out of 177 patients who received inotropes during this period (Vidlund et al., 2016).

The manuscript adheres to the STROBE guidelines.

### Study endpoints

The primary endpoints were PHF and severe PHF according to study criteria. Secondary endpoints were variables associated with PHF: use of inotropes and mechanical circulatory support, myocardial injury, acute kidney injury, duration of ventilator treatment and ICU stay, and hospital mortality.

### NT-proBNP

Sampling for NT-proBNP was done at three time points: immediately before induction of anesthesia, the first and third morning after surgery. Venous blood was collected in lithium heparin tubes and NT-proBNP was measured with electro-chemoiluminescence immunoassay on a Roche Elecsys 2010/ Modular E170 automated platform (Roche Diagnostics, Basel, Switzerland. The assay had an effective measuring range of 5–35,000 ng/L. The inter-assay coefficient of variation was at 175 ng/L CV = 2.7%, 355 ng/L CV = 2.4%, and 1068 ng/L CV = 1.9%. The following upper reference limits (URLs) were applied: 450 ng/L for < 50 years, 900 ng/L for 50–75 years, and 1800 ng/L for > 75 years. Values < 300 ng/L were considered normal in all age groups and the intervals between 300 ng/L and the URL for the age group were considered a grey zone (Maisel et al., 2008; Roberts et al., 2015).

### Clinical management

Clinical management was standardized and similar at the three participating centers with minor differences concerning choice of anesthetic drugs. Standard surgical techniques were used. In 370 patients, standard use of cardiopulmonary bypass (CPB) and aortic cross-clamping was employed. Cold blood cardioplegia was used for myocardial protection in 78% of the patients, whereas crystalloid cardioplegia was used in the remaining patients operated on pump. Twelve patients were operated off pump. A surgical pulmonary artery catheter was introduced intraoperatively in all patients for intermittent measurement of mixed venous oxygen saturation and continuous monitoring of pulmonary artery pressure (Svedjeholm et al., 1999). Mixed venous oxygen saturation (together with arterial blood gases) were measured in all patients after weaning from CPB, 5 min after administration of protamine, on admission to ICU, the next postoperative morning and whenever warranted by the hemodynamic state. Arterial blood pressure, central venous pressure, and pulse oximetry were continuously monitored. Transesophageal echocardiography was routinely employed.

After discharge from the ICU, patients were transferred to a step-down semi-intensive care unit for at least 24 h before going to the general ward. Further details on clinical management can be found in the Additional file.

### Definitions

#### Postoperative heart failure

Patients were considered to have PHF if criteria a+b were fulfilled.

- Decision reached by the Endpoints committee that heart failure was evident at weaning from cardiopulmonary bypass or during the early hours after surgery based on criteria below and supported by available clinical records, echocardiography and hemodynamic data.
- SvO_2_ criteria in relation to SAP that could not be explained by shivering, anemia or hypovolemia. The criteria were based on extensive studies on SvO_2_ with regard to outcome and clinical experience regarding the approximate relationship between SvO_2_ and SAP while using fast acting vasodilator nitroprusside (Holm et al., 2011; Holm et al., 2010; Svedjeholm et al., 1999; Svedjeholm et al., 2010).

$$ {\mathrm{SvO}}_2<50\%,\mathrm{SAP}<130\ \mathrm{mmHg} $$

$$ {\mathrm{SvO}}_2<55\%,\mathrm{SAP}<110\ \mathrm{mmHg} $$

$$ {\mathrm{SvO}}_2<60\%,\mathrm{SAP}<90\ \mathrm{mmHg} $$

$$ {\mathrm{SvO}}_2<65\%,\mathrm{SAP}<70\ \mathrm{mmHg} $$

#### Severe postoperative heart failure

Severe postoperative heart failure was defined as PHF associated with death or requiring treatment with intra-aortic balloon pump or need for at least one inotropic agent in dosages listed in the supplement ≥ 24 h after admission to ICU in patients requiring extended ICU stay (≥ 48 h). Further details are given in the Supplemental methods.

Definitions for preoperative left ventricular dysfunction, postoperative myocardial injury, hospital mortality, acute kidney injury, and postoperative stroke are given in the Supplemental methods.

### Statistical analysis

Categorical variables are presented as percentages and continuous variables as means ± standard deviations. Data that were not normally distributed are expressed as medians (interquartile range). To minimize data loss, missing data were managed with pairwise deletion when possible. Categorical data were compared with Fisher’s exact test. For continuous variables not following a normal distribution, Mann-Whitney *U* test and Wilcoxon signed ranks test were used as appropriate. Kruskal-Wallis test or Pearson Chi-Square Test were used for multiple group comparisons depending on the distribution and nature of data.

Postoperative NT-proBNP was log_10_ transformed before linear regression analysis because of its skewed distribution. To assess the role of PHF or severe PHF on postoperative NT-proBNP levels, a multivariable linear regression model with regard to log_10_NT-proBNP was used, adjusting for possible interaction of glutamate treatment and known non-cardiac confounders age, renal function, gender, and obesity (Redfield et al., 2002; Wang et al., 2004; Chenevier-Gobeaux et al., 2005).

A receiver operating characteristic (ROC) analysis was carried out to calculate the area under the curve (AUC) to evaluate discrimination of postoperative NT-proBNP and its trends with regard to PHF and severe PHF respectively. Youden’s index was used for calculation of best cut-off points with regard to sensitivity and specificity.

Multivariable logistic regression was used to analyze predictors for PHF. Clinically relevant variables and variables with a *p* < 0.25 in the univariable analysis were tested in the model. Hosmer-Lemeshow goodness-of-fit statistics were calculated for the final model.

Statistical analyses were performed with SPSS statistics version 23 (IBM) for windows and Statistica 12.0, StatSoft Inc., Tulsa, OK.

## Results

There were a total of 382 consenting patients with acute coronary syndrome undergoing isolated first-time CABG with at least one available NT-proBNP as follows: preoperative (*n* = 366), postoperative day 1 (POD1; *n* = 320), and postoperative day 3 (POD3; *n* = 325) and data from all three time points available in 267 patients. Preoperative, intraoperative, and postoperative characteristics of the 382 patients are presented in Table 1 and Table 2.

**Table 1:** Preoperative characteristics in all patients, patients without PHF, with PHF, and with severe PHF

Variables	All patients (*n* = 382)	Without PHF (*n* = 347)	PHF (*n* = 35)	*p* value^a^	Severe PHF (*n* = 7)	*p* value^b^
Age (years)	69 [62–75]	68 [62–75]	73 [68–78]	0.004	75 [72–78]	0.035
Female gender	19% (73)	19% (66)	20% (7)	0.82	29% (2)	0.62
BMI (kg/m^2^)	27 [24–30]	27 [24–29]	28 [24–31]	0.39	30 [23–31]	0.78
EuroSCORE II	2.4 [1.6–3.9]	2.3 [1.6–3.8]	3.8 [2.6–6.1]	< 0.0001	9.0 [4.2–11.1]	0.001
Diabetes	24% (91)	23% (79)	34% (12)	0.15	57% (4)	0.06
Hypertension	61% (231)	60% (207)	69% (24)	0.37	100% (7)	0.046
COPD	7% (26)	6% (21)	14% (5)	0.08	14% (1)	0.37
Hemoglobin (g/L)	137 ± 14	137 ± 13	133 ± 14	0.11	121 ± 11	0.002
Troponin T (ng/L)	0 [0–60]	0 [0–50]	30 [0–180]	0.13	220 [30–450]	0.019
p-Creatinine (μmol/L)	91 [80–104]	90 [79–104]	96 [86–115]	0.016	100 [95–147]	0.09
eGFR (mL min^−1^ • 1.73 m^−2^)	76 [58–97]	77 [59–97]	67 [47–84]	0.023	56 [40–79]	0.12
NT-proBNP (ng/L)	420 [150–970]	380 [140–864]	900 [410–1730]	< 0.0001	1920 [1030–4202]	0.001
Cerebrovascular disease	8% (32)	9% (30)	6% (2)	0.75	0	1
Three-vessel disease	77% (293)	75% (260)	94% (33)	0.01	100% (7)	0.2
Left main stenosis	36% (138)	35% (121)	48% (17)	0.14	57% (4)	0.25
AMI < 3 weeks	65% (249)	63% (220)	83% (29)	1	86% (6)	0.43
History of AMI	73% (278)	71% (247)	89% (31)	0.028	86% (6)	0.68
CCS IV	60% (230)	60% (207)	66% (23)	0.59	86% (6)	0.25
Angina at rest < 48 h	17% (61)	16% (53)	24 (8)	0.33	43% (3)	0.09
Moderate LV dysfunction	12% (47)	11% (39)	23% (8)	0.06	43% (3)	0.039
Severe LV dysfunction	4% (14)	3% (9)	14% (5)	0.005	43% (3)	0.001

Data given as medians [interquartile range], percentages (number)

*AMI < 3 weeks* acute myocardial infarction within 3 weeks of surgery, *Angina at rest < 48 h* angina at rest within the last 48 h before surgery, *BMI* body mass index, *CCS IV* Canadian Cardiovascular Society, *COPD* chronic obstructive pulmonary disease, *eGFR* estimated glomerular filtration rate according to MDRD formula, *EuroSCORE II* European system for cardiac operative risk evaluation II, *LV* left ventricular

^a^*p* for without PHF vs. PHF

^b^*p* for without PHF vs. severe PHF

**Table 2:** Intraoperative and postoperative characteristics in all patients, patients with/without PHF, and with severe PHF

Variables	All patients (*n* = 382)	Without PHF (*n* = 347)	PHF (*n* = 35)	*p* value^a^	Severe PHF (*n* = 7)	*p* value^b^
Aortic crossclamp time (min)	49 [39–62]	48 [38–61]	57 [50–67]	0.006	55 [42–67]	0.21
CPB time (min)	75 [62–94]	73 [60–91]	99 [76–118]	< 0.0001	135 [85–150]	0.003
Reperfusion time (min)	20 [15–28]	19 [15–27]	30 [21–42]	< 0.0001	37 [26–56]	0.006
NT-proBNP POD1 (ng/L)	2065 [1324–3650]	2040 [1260–3440]	3240 [1990–5240]	< 0.0001	5040 [3060–10200]	0.001
NT-proBNP POD3 (ng/L)	3610 [2167–6010]	3450 [2003–5550]	6585 [3140–12300]	< 0.0001	11680 [6070–17914]	0.012
Delta NT-proBNP POD1-Pre (ng/L)	1575 [1016–2698]	1515 [941–2510]	2430 [1530–3470]	0.004	3120 [2030–7290]	0.044
Delta NT-proBNP POD3-Pre (ng/L)	3020 [1585–4947]	2857 [1565–4720]	4840 [2818–10520]	0.002	10390 [455–15487]	0.051
Delta NT-proBNP POD3-POD1 (ng/L)	1170 [375–2690]	1140 [350–2500]	2125 [779–5650]	0.028	7060 [− 3050–9702]	0.21
CK-MB POD1(μg/L)	14 [10–22]	14 [9–21]	22 [12–35]	< 0.0001	24 [19–51]	0.014
Troponin T POD3 (ng/L)	250 [140–490]	230 [130–420]	590 [350–1100]	< 0.0001	870 [600–1100]	0.001
Delta Troponin T POD3-Pre (ng/L)	180 [90–370]	170 [85–340]	520 [285–1045]	< 0.0001	570 [520–1100]	0.022
ICU stay (hours)	21 [17–23]	20 [17–23]	41 [21–93]	< 0.0001	122 [47–191]	< 0.0001
ICU stay > 72 h	5% (19)	2% (7)	34% (12)	< 0.0001	71% (5)	< 0.0001
Ventilation time (h)	4 [3–6]	4 [3–5]	8 [6–23]	< 0.0001	49 [6–164]	0.011
Ventilation time > 48 h	3% (11)	1% (4)	20% (7)	< 0.0001	57% (4)	< 0.0001
Postoperative stroke	1% (5)	1% (5)	0	1	0	0.95
AKI	14% (55)	12% (42)	37% (13)	< 0.0001	86% (6)	< 0.0001
Hospital Mortality	1% (5)	1% (2)	9% (3)	0.006	43% (3)	< 0.0001

Data given as medians [interquartile range], percentages (number) or means ± standard deviation. *AKI* acute kidney injury, *CK*-*MB* creatine kinase-MB isoenzyme, *CPB* cardiopulmonary bypass, *ICU* intensive care unit, *POD* postoperative day

^a^*p* for without PHF vs. PHF

^b^*p* for without PHF vs. severe PHF

Overall NT-proBNP increased from 420 [150–970] ng/L preoperatively to 2065 [1324–3650] ng/L (*p* < 0.001) POD1 and to 3610 [2167–6010] ng/L (*p* < 0.001) POD3.

Overall, 88 patients (23%) were treated with inotropes at some stage intraoperatively or postoperatively (Additional table S1). Only 33 of these patients fulfilled criteria for PHF and these patients had significantly worse outcome and more pronounced increase of NT-proBNP postoperatively (Table S1).

Overall, 35 patients (9%) from the whole cohort fulfilled criteria for PHF. Seven of these patients were also classified to have severe PHF. In two patients who fulfilled criteria for PHF, myocardial dysfunction was mild and resolved without inotropes.

Patients with PHF had a more pronounced risk profile preoperatively and extended cross-clamp and CPB times intraoperatively. Postoperatively, they had more signs of myocardial injury, higher incidence of acute kidney injury, extended ventilation time, prolonged ICU stay, and a higher hospital mortality compared to those without PHF. Clinical outcomes were further aggravated in patients with severe PHF (Table 2).

### Postoperative NT-proBNP in relation to PHF

**Fig. 1:**
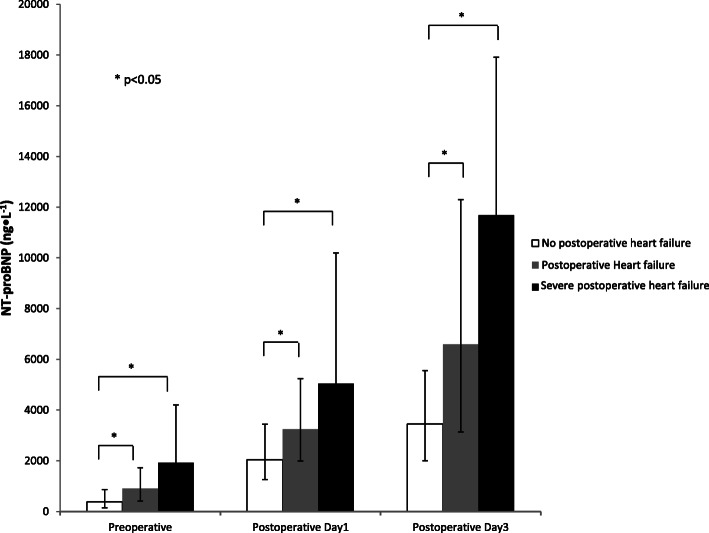
Perioperative NT-proBNP levels in patients without postoperative heart failure (PHF), with PHF and severe PHF. Data given as medians with interquartile range. Mann-Whitney *U* test was performed and *p* < 0.05 was considered significant, indicated by*

Patients with PHF had higher pre- and postoperative levels of NT-proBNP compared to those without PHF (Table 1, Fig. 1).

After adjusting for glutamate treatment and known preoperative non-cardiac confounders age, eGFR, female gender, and obesity, NT-proBNP POD1 was 1.46 times higher in patients with PHF than in patients without PHF (adjusted coefficient 0.165, 95%CI 0.062–0.269, *p* = 0.002; Additional table S2). Interaction of glutamate was not statistically significant and would have changed the adjusted coefficient for PHF by 3% if kept in the final model.

After similar adjustment for glutamate treatment and known preoperative non-cardiac confounders, NT-proBNP POD3 was 1.54 times higher in patients with PHF than in patients without PHF (adjusted coefficient 0.188, 95%CI 0.188–0.289, *p* < 0.0001; Additional table S3). Interaction of glutamate was not statistically significant and would not have changed the adjusted coefficient for PHF if kept in the final model.

**Fig. 2:**
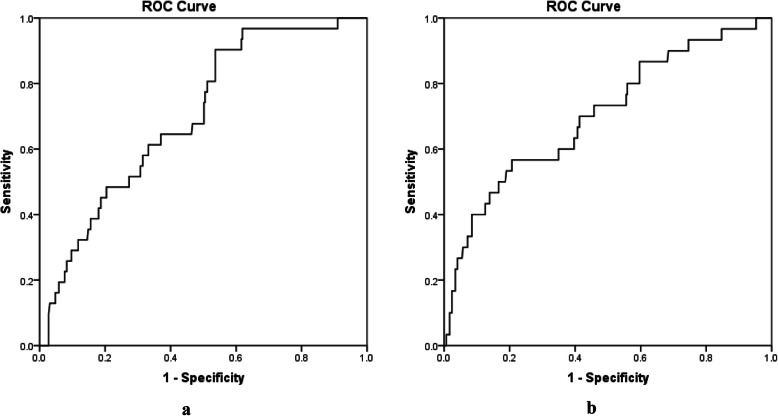
Receiver operating characteristics (ROC) to evaluate discrimination of postoperative NT-proBNP for PHF. Left panel (**a**) demonstrates discrimination of NT-proBNP on POD1 for PHF (AUC = 0.70; 95% CI 0.61–0.79; *p* < 0.0001, best cut-off 1836 ng/L with a sensitivity of 90% and a specificity of 46%, *n* = 320). Right panel (**b**) demonstrates discrimination of NT-proBNP on POD3 for PHF (AUC = 0.70; 95% CI 0.60–0.81; *p* < 0.0001, best cut-off 6065 ng/L with a sensitivity of 57% and a specificity of 79%, *n* = 325). *AUC* area under curve, *CI* confidence interval, *POD1* postoperative day 1, *POD3* postoperative day 3

NT-proBNP on POD1 demonstrated significant discrimination for PHF (AUC 0.70; 95% CI 0.61–0.79; *p* < 0.0001). The best cut-off value of 1836 ng/L had a sensitivity of 90% and a specificity of 46% (Fig. 2a). A similar discrimination was found for NT-proBNP on POD3 (AUC 0.70; 95% CI 0.60–0.81; *p* < 0.0001). The best cut-off value 6065 ng/L had a sensitivity of 57% and a specificity of 79% (Fig. 2b).

### Postoperative changes of NT-proBNP

NT-proBNP increased postoperatively in almost all patients with the highest values recorded on POD3 (Table 1, Fig. 1). The postoperative increase of NT-proBNP was significantly more pronounced in patients with PHF and the postoperative changes of NT-proBNP were associated with PHF.

Postoperative increase of NT-proBNP from preoperative level to POD3 demonstrated significant discrimination for PHF (AUC 0.68; 95% CI 0.56–0.79; *p* = 0.002, best cut-off 7639 ng/L with a sensitivity of 40% and a specificity of 92%). Similar discrimination was found for postoperative change of NT-proBNP from preoperative level to POD1 (AUC 0.66, 95% CI 0.56–0.76, *p* = 0.004, best cut-off 1372 ng/L with a sensitivity of 87% and a specificity of 46%) and for postoperative change of NT-proBNP from POD1 to POD3 (AUC 0.63; 95% CI 0.50–0.76, *p* = 0.028, best cut-off 4299 ng/L with a sensitivity of 38% and a specificity of 90%).

In the multivariable logistic regression analysis, delta Troponin T POD3-Pre ng/L, delta NT-proBNP POD3-POD1 ≥ 4299 ng/L, and severe LV dysfunction emerged as independent risk factors for PHF (Table 3). The univariable Odds ratios for variables tested in are shown in Additional table S4.

**Table 3:** Multivariable analysis^a^ of risk factors for PHF

Variable	Odds ratio	95%CI	*p* value
Delta Troponin T POD3-Pre (ng/L)	1.001	1.000–1.002	0.002
Delta NT-proBNP POD3-POD1 ≥ 4299 ng/L	5.12	1.86–14.10	0.002
Severe LV dysfunction	12.77	2.76-58.99	0.001

Patients with NT-proBNP data from all three time points and aotic cross clamp time available were included in this model (*n* = 257). ^a^Multivariable backward stepwise logistic regression model. Nagelkerke *R*^2^ = 0.28; Hosmer-Lemeshow goodness-of-fit test *x*^2^ (df = 8) = 5.74, *p* = 0.68. *CI* confidence interval

### Postoperative NT-proBNP in relation to severe PHF

The highest pre- and postoperative NT-proBNP values were recorded in patients with severe PHF (Table 1, Fig. 1).

Patients with severe PHF had significantly higher NT-proBNP preoperatively (1920 [1030–4202] v 750 [300–1265] ng/L, *p* = 0.022) and on POD1 (5040 [3060–10,200] v 2740 [1875–4600] ng/L, *p* = 0.028) compared to patients with PHF that was not classified as severe.

Patients with severe PHF had significantly higher NT-proBNP preoperatively (1920 [1030–4202] v 380 [140–864] ng/L, *p* = 0.001), on POD1(5040 [3060–10,200] v 2040 [1260–3440] ng/L, *p* = 0.001 and on POD3 (11680 [6070–17,914] v 3450 [2003–5550] ng/L, *p* = 0.012) compared to patients without PHF.

After adjusting for glutamate treatment and known preoperative non-cardiac confounders age, eGFR, female gender, and obesity, NT-proBNP POD1 was 2.18 times higher in patients with severe PHF than in patients without PHF (adjusted coefficient 0.339, 95%CI 0.134–0.543, *p* = 0.001; Additional table S5). Interaction of glutamate was not statistically significant would have changed the adjusted coefficient for severe PHF by 6% if kept in the final model.

After similar adjustment for glutamate treatment and known preoperative non-cardiac confounders, NT-proBNP POD3 was 1.81 times higher in patients with severe PHF than in patients without PHF (adjusted coefficient 0.258, 95%CI 0.042–0.474, *p* = 0.019; Additional table S6). Interaction of glutamate was not statistically significant would have changed the adjusted coefficient for severe PHF by 0.3% if kept in the final model.

**Fig. 3:**
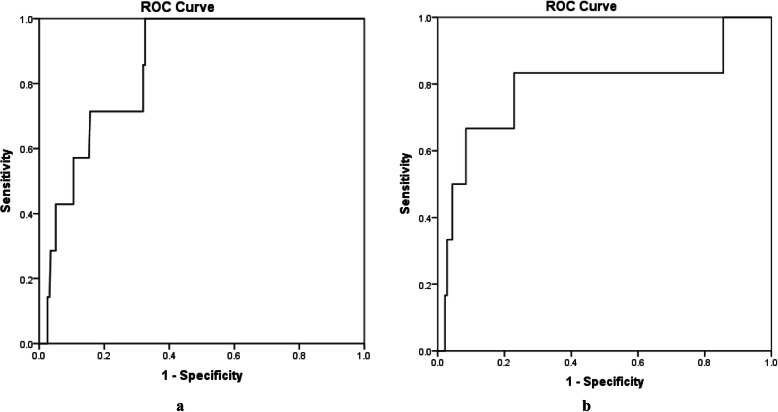
Receiver operating characteristics (ROC) to evaluate discrimination of postoperative NT-proBNP for severe PHF. Left panel (**a**) demonstrates discrimination of NT-proBNP on POD1 for severe PHF (AUC = 0.86; 95% CI 0.76–0.95; *p* = 0.001, best cut-off 4574 ng/L with a sensitivity of 71%, *n* = 320). Right panel (**b**) demonstrates discrimination of NT-proBNP on POD3 for severe PHF (AUC = 0.79; 95% CI 0.55–1.00; *p* = 0.015, best cut-off 6065 ng/L with a sensitivity of 83% and a specificity of 77%, *n* = 325). *AUC* area under curve, *CI* confidence interval, *POD1* postoperative day 1, *POD3* postoperative day 3

NT-proBNP on POD1 demonstrated significant discrimination for severe PHF (AUC = 0.86; 95% CI 0.76–0.95; *p* = 0.001). The best cut-off value of 4575 ng/L had a sensitivity of 71% and a specificity of 84% (Fig. 3a). A similar discrimination was found for NT-proBNP on POD3 (AUC = 0.79; 95% CI 0.55–1.00; *p* = 0.015) (Fig. 3b). The best cut-off value of 6065 ng/L had a sensitivity of 83% and a specificity of 77%.

The number of events was too few to permit multivariable analysis of risk factors for severe PHF.

Patients with NT-proBNP above the cut-offs had more pronounced Troponin T elevations, higher incidence of acute kidney injury, extended ventilation time, prolonged ICU stay, and higher hospital mortality (Supplemental tables S7-S8).

### Circulatory support and variables associated with PHF in incremental quartiles of postoperative NT-proBNP

The incidence of inotropic treatment, the use of more than one drug, and the average duration of treatment was higher in patients with NT-proBNP POD1 and POD3 in the upper quartiles. Extended ICU stay and ventilator support, and renal dysfunction, were also more common in the upper quartiles of postoperative NT-proBNP levels.

Patients with PHF and severe PHF belonged to the upper quartiles of postoperative NT-proBNP. A notable exception was one patient who was in severe heart failure preoperatively. This patient improved markedly after surgery and despite fulfilling study criteria for severe PHF plasma levels of NT-proBNP decreased from 9250 ng/L preoperatively to 4640 ng/L POD1 and 1590 ng/L POD3 (lowest quartile).

Detailed results regarding circulatory support and postoperative outcome in incremental quartiles of postoperative NT-proBNP levels are presented in Tables 4 and 5.

**Table 4:** Circulatory support and postoperative outcome related to incremental quartiles of NT-proBNP on POD1

	NT-proBNP POD1 < 1324 ng/L (*n* = 80)	NT-proBNP POD1 ≥ 1324 ng/L to < 2065 ng/L (*n* = 80)	NT-proBNP POD1 ≥ 2065 ng/L to < 3650 ng/L (*n* = 80)	NT-proBNP POD1 ≥ 3650 ng/L (*n* = 80)	*p* value
PHF	1% (1)	11% (9)	8% (6)	19% (15)	0.002
Severe PHF	0	0	3% (2)	6% (5)	0.02
Use of Inotrope	10% (8)	20% (16)	23% (18)	43% (34)	< 0.0001
Use of IABP	0	0	1% (1)	1% (1)	0.57
Duration of inotropic treatment ^a^(h)	1.0 ± 3.9	6.3 ± 20.4	9.0 ± 26.4	19.1 ± 42.5	< 0.0001
Duration of inotropic treatment ^b^(h)	10.1 ± 8.1	31.6 ± 36.6	39.9 ± 44.0	44.9 ± 56.0	0.21
Duration of inotropic treatment > 24 h	0	6% (5)	11% (9)	20% (16)	< 0.0001
More than one Inotrope at the same time	1% (1)	3% (2)	5% (4)	10% (8)	0.045
Adrenalin used	9% (7)	18% (14)	21% (17)	33% (26)	0.002
Milrinone used	4% (3)	8% (6)	8% (6)	28% (14)	0.016
Levosimendan used	0	0	1% (1)	5% (4)	0.031
Troponin T POD3 (ng/L)	180 [110–280]	250 [140–355]	220 [130–600]	450 [200–725]	< 0.0001
Delta Troponin T POD3-Pre (ng/L)	140 [58–230]	190 [110–350]	170 [80–430]	300 [135–520]	0.001
ICU stay (h)	19.1 ± 10.5	22.8 ± 16.7	23.5 ± 21.5	53.1 ± 101.1	0.002
ICU stay > 72 h	0	4% (3)	3% (2)	13% (10)	0.001
Ventilation time (h)	4.4 ± 4.5	5.3 ± 5.9	6.7 ± 18.1	28.9 ± 93.2	0.001
Ventilation time > 48 h	0	0	1% (1)	10% (8)	< 0.0001
AKI	8% (6)	4% (3)	13% (10)	30% (24)	< 0.0001
Postoperative stroke	1% (1)	0	1% (1)	1% (1)	0.8
Hospital mortality	0	0	3% (2)	4% (3)	0.14

Data given as medians [interquartile range], percentages (number) or means ± standard deviation. ^a^Average duration of inotropic treatment including all patients. ^b^Average duration of inotropic treatment in those receiving inotropic treatment. *AKI* acute kidney injury, *CK*-*MB* creatine kinase-MB isoenzyme, *ICU* intensive care unit, *POD* postoperative day

**Table 5:** Circulatory support and postoperative outcome related to incremental quartiles of NT-proBNP on POD3

	NT-proBNP POD3< 2167 (*n* = 81)	NT-proBNP POD3≥2167 to <3610(*n* = 81)	NT-proBNP POD3≥3610 to <6010 (*n* = 81)	NT-proBNP POD3≥6010 (*n* = 82)	*p* value
PHF	4% (3)	6% (5)	6% (5)	21% (17)	0.001
Severe PHF	1% (1)	0	0	6%(5)	0.01
Use of Inotrope	11% (9)	22% (18)	23% (19)	39% (32)	0.001
Use of IABP	0	0	0	2% (2)	0.11
Duration of inotropic treatment^a^ (h)	3.2 ± 16.9	7.2 ± 25.7	6.0 ± 14.9	18.7 ± 41.9	0.004
Duration of inotropic treatment ^b^(h)	29.2 ± 44.8	32.6 ± 47.3	25.5 ± 21.3	47.9 ± 56.1	0.61
Duration of inotropic treatment > 24 h	2% (2)	6% (5)	9% (7)	20% (16)	0.001
More than one Inotrope at the same time	4% (3)	2% (2)	4% (3)	11% (9)	0.06
Adrenalin used	11% (9)	19% (15)	21% (17)	32% (26)	0.011
Milrinone used	5% (4)	7% (6)	7% (6)	19% (15)	0.016
Levosimendan used	1% (1)	0	1% (1)	6% (5)	0.035
Troponin T POD3 (ng/L)	180 [100–295]	210 [120–370]	250 [130–455]	490 [230–750]	< 0.0001
Delta Troponin T POD3-Pre (ng/L)	135 [35-220]	190 [100-355]	170 [90-340]	390 [175-600]	< 0.0001
ICU stay (h)	18.2 ± 11.3	30.4 ± 55.7	22.5 ± 15.6	48.7 ± 88.6	< 0.0001
ICU stay > 72 h	1% (1)	5% (4)	2% (2)	11% (9)	0.02
Ventilation time (h)	4.0 ± 5.3	12.3 ± 52.2	5.1 ± 5.0	25.9 ± 81.4	< 0.0001
Ventilation time > 48 h	1% (1)	2% (2)	0	9% (7)	0.008
AKI	9% (7)	10% (8)	6% (5)	32% (26)	< 0.0001
Postoperative stroke	0	1% (1)	0	4%(3)	0.11
Hospital mortality	0	0	0	5% (4)	0.007

Data given as medians [interquartile range], percentages (number), or means ± standard deviation. ^a^Average duration of inotropic treatment including all patients. ^b^Average duration of inotropic treatment in those receiving inotropic treatment. *AKI* acute kidney injury, *CK*-*MB* creatine kinase-MB isoenzyme, *ICU* intensive care unit, *POD* postoperative day

## Discussion

Previous studies show that PHF is associated with increased levels of natriuretic peptides postoperatively (Fox et al., 2008; Kerbaul et al., 2004; Reyes et al., 2005; Nozohoor et al., 2009; Suttner et al., 2008; Provenchere et al., 2006). The results of this study confirm these findings and add to the knowledge by showing that this is true also after adjusting for non-cardiac variables known to influence NT-proBNP, and that the levels of NT-proBNP reflect the severity of PHF. Both absolute postoperative levels and postoperative changes of NT-proBNP were associated with PHF.

To our knowledge, this is the first prospective study in which a blinded assessment of postoperative NT-proBNP with regard to PHF and its severity was done relying on prespecified hemodynamic criteria. It is also the first study on natriuretic peptides and PHF to adjust for non-cardiac variables known to influence plasma levels.

Reliance on treatment criteria, such as need of inotropes or mechanical cardiac assist devices, as criteria for PHF are clouded by the large differences between geographical regions, institutions, and individuals regarding threshold for institution of treatment or prophylaxis (Fellahi et al., 2008; Mehta et al., 2017). In our study, just over one-third of patients treated with inotropes fulfilled hemodynamic criteria for PHF and these patients had significantly worse outcome and more pronounced increase of NT-proBNP postoperatively. The hemodynamic criteria used by the blinded endpoints committee were mainly based on SvO_2_ measurements accounting for non-cardiac parameters such as anemia, hypovolemia, and shivering that impact SvO_2_ measurements (Holm et al., 2011; Holm et al., 2010; Svedjeholm et al., 1999).

The elevated postoperative levels of NT-proBNP were caused by higher plasma concentrations preoperatively and more pronounced increases postoperatively. Multivariable analysis identified left ventricular ejection fraction ≤ 0.30, intraoperative myocardial injury, and postoperative increase of NT-proBNP to be associated with PHF. Postoperative NT-proBNP, thus, seems to reflect the preoperative condition of the heart as well as myocardial dysfunction sustained during and after surgery. This is in agreement with the literature, which reports preoperative left ventricular dysfunction and perioperative myocardial infarction to be important causes for PHF in patients undergoing CABG (Vanky et al., 2004; Algarni et al., 2011). In accordance with previous studies, PHF and in particular severe PHF was associated with increased postoperative morbidity and mortality (O'Connor et al., 1998; Surgenor et al., 2001; Vanky et al., 2004; Rao et al., 1996).

NT-proBNP on POD1 and POD3 displayed a significant and acceptable discrimination for PHF and a good discrimination for severe PHF. Given that the group with PHF included some mild cases of heart failure, it is not surprising that the discrimination of NT-proBNP was more evident with regard to severe PHF. The results and cut-off levels in particular should be interpreted with caution due to the low number of patients with severe PHF but they suggest that postoperative NT-proBNP reflects the severity of PHF.

To further address this issue and to shed more light on the spectrum of postoperative NT-proBNP levels, we scrutinized the use of circulatory support. Use of inotropic drugs was therefore described in greater detail than previously reported on this topic and the results clearly show that with incremental levels of plasma NT-proBNP the use of inotropes increases regarding proportion of patients treated, number of drugs used, and the duration of treatment. Increased incidence of PHF and severe PHF, extended ICU stay and ventilator support, and renal dysfunction were also observed with incremental levels of postoperative NT-proBNP levels.

All in all, the results indicate that NT-proBNP can serve as a marker for the degree of postoperative myocardial dysfunction and PHF. This is not unexpected given that the release of B-type natriuretic peptide into the circulation is proportional to the ventricular expansion and volume overload of the ventricles and therefore reflects the decompensated state of the ventricles (Daniels & Maisel, 2007; Maeda et al., 1998). However, it is also evident that there is a substantial overlap in plasma levels between patients with and without PHF and that a large proportion of patients in the highest quartile of postoperative NT-proBNP did not fulfill criteria for PHF or need treatment with inotropes.

The overlap between patients with and without heart failure is partly explained by non-cardiac factors such as age, renal function, gender, obesity, and inflammation, which are known to influence the plasma levels of natriuretic peptides. In cardiology practice, reference values for NT-proBNP have been adjusted for age and include a wide grey zone between what is considered normal (rule out) and abnormal (Maisel et al., 2008). We did not have data on inflammation but after adjusting for glutamate treatment, age, eGFR, female gender, and obesity we found that NT-proBNP on average was approximately 1.5 times and 2 times higher postoperatively in patients with PHF and severe PHF respectively on POD1 and POD3.

Due to the overlap in postoperative NT-proBNP levels between patients with or without PHF, the test is obviously not suited for comparing individual patients. However, the results clearly show that a cohort with higher levels of NT-proBNP has a more pronounced degree of postoperative myocardial dysfunction on average than a cohort with lower levels of NT-proBNP. This implies that NT-proBNP could serve as an objective marker of postoperative myocardial dysfunction, and thus be useful for scientific purposes evaluating treatment and prevention of PHF. However, such studies need to account for known confounders and if applicable to stratify for type of procedure as underlying pathophysiology will influence natriuretic peptide levels (Jiang et al., 2018).

In cardiology practice, current guidelines recommend the use of natriuretic peptides as the first line biomarkers for the diagnosis, prognosis, and follow-up of patients with heart failure (Ponikowski et al., 2016; Yancy et al., 2017). Patients responding to treatment with a reduction of natriuretic peptides have a better prognosis than non-responders (Karlstrom et al., 2011; Felker et al., 2009). Our study also suggests that postoperative trends of NT-proBNP are important. The latter is in conflict with a study by Mauermann et al, who found postoperative changes of BNP to be unimportant (Mauermann et al., 2017). However, Mauermann studied change of BNP from POD1 to POD2, which may have been too short given that BNP normally peaks on POD3-4 (Mauermann et al., 2017). Furthermore, the endpoint in Mauermanns study was all-cause mortality 12 months after surgery.

In clinical practice, echocardiography and hemodynamic monitoring with pulmonary artery catheters obviously provide information important for detection of PHF and its management, but they may not be as suitable for large clinical trials. In contrast to pulmonary artery catheter measurements (rarely used routinely) and transesophageal echocardiography (investigator dependent) natriuretic peptides provide a readily accessible and inexpensive option for objective assessment of PHF in clinical trials.

Some limitiations deserve consideration. Data on other potential confounders such as markers of inflammation and pharmacological treatment would have been desirable. Although the number of patients is larger than in most previous studies on postoperative natriuretic peptides, the cut-off levels and odds ratios in our study should be interpreted cautiously due to the limited number of events. To maintain sample size, patients treated with intravenous glutamate infusion were not excluded as interaction according multivariable analysis was limited. Patients operated off-pump were also included as they did not differ significantly from CPB patients regarding NT-proBNP. Finally, it should be emphasized that the results were obtained in patients undergoing isolated first-time CABG and, hence, are not applicable to other cardiac surgical patients.

## Conclusions

Postoperative NT-proBNP can serve as an objective marker of the severity of postoperative myocardial dysfunction after CABG but due to overlap in individuals, NT-proBNP is mainly useful for comparisons at cohort level. As such, it provides a tool for study purposes when an unbiased assessment of prevention or treatment of PHF is desirable.

## Supplementary Information

**Additional file 1: Supplemental methods**. **Tables S1.** Pre- and postoperative data in patients treated with inotropes depending on if they fulfilled criteria for PHF diagnosis or not. **Table S2.** Multivariable linear regression results for log_10_ NT-proBNP POD1 in all patients adjusted for PHF, glutamate treatment and known preoperative non-cardiac confounders. **Table S3.** Multivariable linear regression results for log_10_ NT-proBNP POD3 in all patients adjusted for PHF, glutamate treatment and known preoperative non-cardiac confounders. **Table S4.** Variables associated with PHF according to univariable logistic regression. **Table S5.** Multivariable linear regression results for log_10_ NT-proBNP POD1 adjusted for severe PHF, glutamate treatment and known preoperative non-cardiac confounders. **Table S6.** Multivariable linear regression results for log_10_ NT-proBNP POD3 adjusted for severe PHF, glutamate treatment and known preoperative non-cardiac confounders. **Table S7.** Postoperative data in patients with NT-proBNP POD1<4575ng/L or ≥ 4575ng/L. **Table S8.** Postoperative data in patients with NT-proBNP POD3<6065 ng/L or ≥ 6065ng/L. **Figure S1.** Flow chart of the patients in this substudy of the GLUTAMICS trial.

## Data Availability

The datasets analyzed during the current study are available on reasonable request provided that professional secrecy applies. Qualified researchers may apply for access through the Chief of the Dept. of Cardiothoracic and vascular Surgery, Linköping Heart Center, University Hospital, SE-58185 Linköping, Sweden.

## Abbreviations

AKI: Acute kidney injury
AMI: Acute myocardial infarction
AUC: Area under the curve
BMI: Body mass index
BNP: B-type natriuretic peptide
CABG: Coronary artery bypass surgery
CCS: Canadian Cardiovascular Society
CI: Confidence interval
CK-MB: Creatine kinase-MB isoenzyme
COPD: Chronic obstructive pulmonary disease
CPB: Cardiopulmonary bypass
eGFR: Estimated glomerular filtration rate according to MDRD formula
EuroSCORE II: European system for cardiac operative risk evaluation II
GLUTAMICS: GLUTAmate for Metabolic Intervention in Coronary Surgery trial
ICU: Intensive care unit
LV: Left ventricular
NT-proBNP: N-terminal pro-B-type natriuretic peptide
PHF: Postoperative heart failure
POD: Postoperative day
ROC: Receiver operating characteristic analysis
SAP: Systolic arterial blood pressure
SvO_2_: Mixed venous oxygen saturation
URL: Upper reference limit

## References

[CR1] Algarni KD, Maganti M, Yau TM (2011). Predictors of low cardiac output syndrome after isolated coronary artery bypass surgery: trends over 20 years. Ann Thorac Surg.

[CR2] Breisblatt WM, Stein KL, Wolfe CJ, Follansbee WP, Capozzi J, Armitage JM, Hardesty RL (1990). Acute myocardial dysfunction and recovery: a common occurrence after coronary bypass surgery. J Am Coll Cardiol.

[CR3] Chenevier-Gobeaux C, Claessens YE, Voyer S, Desmoulins D, Ekindjian OG (2005). Influence of renal function on N-terminal pro-brain natriuretic peptide (NT-proBNP) in patients admitted for dyspnoea in the Emergency Department: comparison with brain natriuretic peptide (BNP). Clin Chim Acta.

[CR4] Coronel R, de Groot JR, van Lieshout JJ (2001). Research ObotetoC. Defining heart failure. Cardiovasc Res.

[CR5] Crescenzi G, Landoni G, Bignami E, Belloni I, Biselli C, Rosica C, Guarracino F, Marino G, Zangrillo A (2009). N-terminal B-natriuretic peptide after coronary artery bypass graft surgery. J Cardiothorac Vasc Anesth.

[CR6] Daniels LB, Maisel AS (2007). Natriuretic peptides. J Am Coll Cardiol.

[CR7] Felker GM, Hasselblad V, Hernandez AF, O’Connor CM (2009). Biomarker-guided therapy in chronic heart failure: a meta-analysis of randomized controlled trials. Am Heart J.

[CR8] Fellahi JL, Parienti JJ, Hanouz JL, Plaud B, Riou B, Ouattara A (2008). Perioperative use of dobutamine in cardiac surgery and adverse cardiac outcome: propensity-adjusted analyses. Anesthesiology.

[CR9] Fox AA, Shernan SK, Collard CD, Liu KY, Aranki SF, DeSantis SM (2008). Preoperative B-type natriuretic peptide is as independent predictor of ventricular dysfunction and mortality after primary coronary artery bypass grafting. J Thorac Cardiovasc Surg.

[CR10] Gillies M, Bellomo R, Doolan L, Buxton B (2005). Bench-to-bedside review: inotropic drug therapy after adult cardiac surgery—a systematic literature review. Crit Care.

[CR11] Hakanson E, Svedjeholm R, Vanhanen I (1995). Physiologic aspects in postoperative cardiac patients. Ann Thorac Surg.

[CR12] Holm J, Hakanson E, Vanky F, Svedjeholm R (2011). Mixed venous oxygen saturation predicts short- and long-term outcome after coronary artery bypass grafting surgery: a retrospective cohort analysis. Br J Anaesth.

[CR13] Holm J, Hakanson RE, Vanky F, Svedjeholm R (2010). Mixed venous oxygen saturation is a prognostic marker after surgery for aortic stenosis. Acta Anaesthesiol Scand.

[CR14] Holm J, Vidlund M, Vanky F, Friberg O, Hakanson E, Walther S (2014). EuroSCORE II and N-terminal pro-B-type natriuretic peptide for risk evaluation: an observational longitudinal study in patients undergoing coronary artery bypass graft surgery. Br J Anaesth.

[CR15] Jiang H, Hultkvist H, Holm J, Vanky F, Yang Y, Svedjeholm R (2018). Impact of underlying heart disease per se on the utility of preoperative NT-proBNP in adult cardiac surgery. PLoS One..

[CR16] Karlstrom P, Alehagen U, Boman K, Dahlstrom U, Group UP-s (2011). Brain natriuretic peptide-guided treatment does not improve morbidity and mortality in extensively treated patients with chronic heart failure: responders to treatment have a significantly better outcome. Eur J Heart Fail.

[CR17] Kerbaul F, Collart F, Giorgi R, Oddoze C, Lejeune PJ, Guidon C, Caus T, Bellezza M, Gouin F (2004). Increased plasma levels of pro-brain natriuretic peptide in patients with cardiovascular complications following off-pump coronary artery surgery. Intensive Care Med.

[CR18] Lurati Buse GA, Koller MT, Burkhart C, Seeberger MD, Filipovic M (2011). The predictive value of preoperative natriuretic peptide concentrations in adults undergoing surgery: a systematic review and meta-analysis. Anesth Analg.

[CR19] Maeda K, Tsutamoto T, Wada A, Hisanaga T, Kinoshita M (1998). Plasma brain natriuretic peptide as a biochemical marker of high left ventricular end-diastolic pressure in patients with symptomatic left ventricular dysfunction. Am Heart J.

[CR20] Maisel A, Mueller C, Adams K, Anker SD, Aspromonte N, Cleland JG (2008). State of the art: using natriuretic peptide levels in clinical practice. Eur J Heart Fail.

[CR21] Mauermann E, Bolliger D, Fassl J, Grapow M, Seeberger EE, Seeberger MD, Filipovic M, Lurati Buse GAL (2017). Absolute postoperative B-type natriuretic peptide concentrations, but not their general trend, are associated with 12-month, all-cause mortality after on-pump cardiac surgery. Anesth Analg.

[CR22] Mehta RH, Leimberger JD, van Diepen S, Meza J, Wang A, Jankowich R, Harrison RW, Hay D, Fremes S, Duncan A, Soltesz EG, Luber J, Park S, Argenziano M, Murphy E, Marcel R, Kalavrouziotis D, Nagpal D, Bozinovski J, Toller W, Heringlake M, Goodman SG, Levy JH, Harrington RA, Anstrom KJ, Alexander JH (2017). Levosimendan in patients with left ventricular dysfunction undergoing cardiac surgery. N Engl J Med.

[CR23] Mitchell J, Webb ST (2011). Is brain natriuretic peptide a marker for adverse postoperative outcomes in patients undergoing cardiac surgery?. Interact Cardiovasc Thorac Surg.

[CR24] Nozohoor S, Nilsson J, Luhrs C, Roijer A, Algotsson L, Sjogren J (2009). B-type natriuretic peptide as a predictor of postoperative heart failure after aortic valve replacement. J Cardiothorac Vasc Anesth.

[CR25] O'Connor GT, Birkmeyer JD, Dacey LJ, Quinton HB, Marrin CA, Birkmeyer NJ (1998). Results of a regional study of modes of death associated with coronary artery bypass grafting. Northern New England Cardiovascular Disease Study Group. Ann Thorac Surg.

[CR26] Ponikowski P, Voors AA, Anker SD, Bueno H, Cleland JG, Coats AJ, Falk V, González-Juanatey JR, Harjola VP, Jankowska EA, Jessup M, Linde C, Nihoyannopoulos P, Parissis JT, Pieske B, Riley JP, Rosano GM, Ruilope LM, Ruschitzka F, Rutten FH, van der Meer P, Authors/Task Force Members, Document Reviewers (2016). 2016 ESC guidelines for the diagnosis and treatment of acute and chronic heart failure: the task force for the diagnosis and treatment of acute and chronic heart failure of the European Society of Cardiology (ESC). Developed with the special contribution of the Heart Failure Association (HFA) of the ESC. Eur J Heart Fail.

[CR27] Provenchere S, Berroeta C, Reynaud C, Baron G, Poirier I, Desmonts JM (2006). Plasma brain natriuretic peptide and cardiac troponin I concentrations after adult cardiac surgery: association with postoperative cardiac dysfunction and 1-year mortality. Crit Care Med.

[CR28] Rao V, Ivanov J, Weisel RD, Ikonomidis JS, Christakis GT, David TE (1996). Predictors of low cardiac output syndrome after coronary artery bypass. J Thorac Cardiovasc Surg.

[CR29] Redfield MM, Rodeheffer RJ, Jacobsen SJ, Mahoney DW, Bailey KR, Burnett JC (2002). Plasma brain natriuretic peptide concentration: impact of age and gender. J Am Coll Cardiol.

[CR30] Reyes G, Fores G, Rodriguez-Abella RH, Cuerpo G, Vallejo JL, Romero C (2005). NT-proBNP in cardiac surgery: a new tool for the management of our patients?. Interact Cardiovasc Thorac Surg.

[CR31] Roberts E, Ludman AJ, Dworzynski K, Al-Mohammad A, Cowie MR, McMurray JJ (2015). The diagnostic accuracy of the natriuretic peptides in heart failure: systematic review and diagnostic meta-analysis in the acute care setting. BMJ.

[CR32] Surgenor SD, O'Connor GT, Lahey SJ, Quinn R, Charlesworth DC, Dacey LJ (2001). Predicting the risk of death from heart failure after coronary artery bypass graft surgery. Anesth Analg.

[CR33] Suttner S, Boldt J, Lang K, Rohm KD, Piper SN, Mayer J (2008). Association of N-terminal pro-brain natriuretic peptide and cardiac troponin T with in-hospital cardiac events in elderly patients undergoing coronary artery surgery. Eur J Anaesthesiol.

[CR34] Svedjeholm R, Hakanson E, Szabo Z (1999). Routine SvO2 measurement after CABG surgery with a surgically introduced pulmonary artery catheter. Eur J Cardiothorac Surg.

[CR35] Svedjeholm R, Vidlund M, Vanhanen I, Hakanson E (2010). A metabolic protective strategy could improve long-term survival in patients with LV-dysfunction undergoing CABG. Scand Cardiovasc J.

[CR36] Vanhanen I, Håkanson E, Jorfeldt L, Svedjeholm R (1998). Intravenous aspartate infusion after a coronary operation: effects on myocardial metabolism and hemodynamic state. Ann Thorac Surg.

[CR37] Vanky F, Hakanson E, Maros T, Svedjeholm R (2004). Different characteristics of postoperative heart failure after surgery for aortic stenosis and coronary disease. Scand Cardiovasc J.

[CR38] Vidlund M, Hakanson E, Friberg O, Juhl-Andersen S, Holm J, Vanky F (2012). GLUTAMICS--a randomized clinical trial on glutamate infusion in 861 patients undergoing surgery for acute coronary syndrome. J Thorac Cardiovasc Surg.

[CR39] Vidlund M, Tajik B, Hakanson E, Friberg O, Holm J, Vanky F (2016). Post hoc analysis of the glutamics-trial: intravenous glutamate infusion and use of inotropic drugs after cabg. BMC Anesthesiol.

[CR40] Wang TJ, Larson MG, Levy D, Benjamin EJ, Leip EP, Wilson PW (2004). Impact of obesity on plasma natriuretic peptide levels. Circulation.

[CR41] Yancy CW, Jessup M, Bozkurt B, Butler J, Casey DE, Colvin MM (2017). 2017 ACC/AHA/HFSA focused update of the 2013 ACCF/AHA guideline for the management of heart failure: a report of the American College of Cardiology/American Heart Association task force on clinical practice guidelines and the Heart Failure Society of America. J Card Fail.

[CR42] Young YR, Sheu BF, Li WC, Hsieh TM, Hung CW, Chang SS (2014). Predictive value of plasma brain natriuretic peptide for postoperative cardiac complications—a systemic review and meta-analysis. J Crit Care.

