# Supplementary material for: Utility of NT-proBNP as an objective marker of postoperative heart failure after coronary artery bypass surgery: a prospective observational study
Source: Perioper Med (Lond). 2021 Jul 13;10:21. doi: 10.1186/s13741-021-00194-4 (PMC8275184; doi:10.1186/s13741-021-00194-4)
Supplement: Supplementary file 1 — Additional file 1: Supplemental methods. Tables S1. Pre- and postoperative data in patients treated with inotropes depending on if they fulfilled criteria for PHF diagnosis or not. Table S2. Multivariable linear regression results for log10 NT-proBNP POD1 in all patients adjusted for PHF, glutamate treatment and known preoperative non-cardiac confounders. Table S3. Multivariable linear regression results for log10 NT-proBNP POD3 in all patients adjusted for PHF, glutamate treatment and known preoperative non-cardiac confounders. Table S4. Variables associated with PHF according to univariable logistic regression. Table S5. Multivariable linear regression results for log10 NT-proBNP POD1 adjusted for severe PHF, glutamate treatment and known preoperative non-cardiac confounders. Table S6. Multivariable linear regression results for log10 NT-proBNP POD3 adjusted for severe PHF, glutamate treatment and known preoperative non-cardiac confounders. Table S7. Postoperative data in patients with NT-proBNP POD1<4575ng/L or ≥ 4575ng/L. Table S8. Postoperative data in patients with NT-proBNP POD3<6065 ng/L or ≥ 6065ng/L. Figure S1. Flow chart of the patients in this substudy of the GLUTAMICS trial. [file 13741_2021_194_MOESM1_ESM.docx]

**ADDITIONAL FILE**

**Utility of NT-proBNP as an objective marker of postoperative heart failure after coronary artery bypass surgery: a prospective observational study**

**SUPPLEMENTAL METHODS**

A prespecified substudy of the GLUTAMICS-trial - GLUTAmate for Metabolic Intervention in Coronary Surgery (ClinicalTrials.gov Identifier: [NCT00489827](http://clinicaltrials.gov/ct2/show/NCT00489827)).

**THE GLUTAMICS TRIAL**

The Glutamics trial was an investigator-initiated prospective randomised controlled trial evaluating metabolic intervention with intravenous glutamate infusion in association with surgery for acute coronary syndrome.^1^

The primary endpoint was a composite of postoperative mortality (≤30 days), perioperative myocardial infarction and left ventricular heart failure in association with weaning from cardiopulmonary bypass or after completion of off-pump coronary artery bypass surgery.

Postoperative mortality and stroke with 24 hours of surgery were safety endpoints.

The study was approved by the Swedish Medical Products Agency (151:2003/70403) and the Regional Ethical Review Board in Linköping (M76-05). Amendments were accepted by the Swedish Medical Products Agency 2006-08-31, 2007-05-08, 2007-11-01 and 2007-11-19.

External randomisation in variable block sizes was done by Apoteket AB, Produktion & Laboratorier (APL), Box 6124, SE 90604, Umeå, Sweden.

External monitoring of all key data was done by an independent professional monitoring team (Clinical Research Support: <http://www.orebroll.se/crs>). Recording of adverse events was done according to Good Clinical Practice standard.

**INTERVENTION**

**Glutamate solution**

500 ml 0.125 M solution of L-glutamic acid with pH 6.0 and 280 mosmol/kg containing L-glutamic acid 9.2 g, NaCl 0.8g, H_2_O ad 500 ml and NaOH quantum satis.

Production of glutamate solution and quality control was done by Apoteket AB, Produktion & Laboratorier (APL), Box 6124, SE 90604 Umeå, Sweden.

**INCLUSION CRITERIA**

Inclusion criteria were coronary artery bypass surgery for acute coronary syndrome. Patients were eligible for inclusion regardless if the procedure was done on-pump or off-pump or if the patient had a simultaneous valve procedure.

**EXCLUSION CRITERIA**

Exclusion criteria were, informed consent not possible because of critical condition or other reason, preoperative use of inotropic drugs or mechanical circulatory assist, preoperative dialysis, redo-procedure, unexpected intraoperative finding or event that increased the magnitude of the procedure to overshadow the originally planned operation, age > 85 years, body weight > 125 kg and food allergy known to have caused flush, rash or asthma.

**CLINICAL ENDPOINTS COMMITTEE**

The clinical endpoints committee consisted of consultants in cardiothoracic surgery and cardiothoracic anaesthesiology from each of the participating centres. The members of the committee were blinded to the treatment assignment and prespecified criteria reported in the manuscript were used to reach a consensus decision. The committee was also blinded to the results of the NT-proBNP analyses.

All cases with suspected postoperative heart failure (PHF) based on SvO_2_ and systemic blood pressure, pulmonary artery pressure, central venous pressure, use of inotropic drugs or mechanical circulatory support, extended ICU stay or circulatory problems reported by the anaesthesiologists or surgeons in the clinical database were reviewed. The committee decided whether circulatory problems that met the prespecified criteria had occurred, if these circulatory problems were severe and if they were cardiac in origin, if they were evident at weaning from cardiopulmonary bypass or presented later in the postoperative course. The committee also decided if events leading to death were cardiac in origin. Pre- and postoperative data in patients treated with inotropes depending on if they fulfilled PHF criteria or not are given in Table S1.

Data on late mortality was retrieved from the Swedish Civil Registry and the cause for late mortality was retrieved from the Causes of Death Registry at the Swedish National Board of Health and Welfare.

**DEFINITIONS**

**Postoperative heart failure**

Patients were considered to have PHF if criteria a+b were fulfilled.

a) Decision reached by the Endpoints committee that heart failure was evident at weaning from cardiopulmonary bypass or during the early hours after surgery based on criteria below and supported by available clinical records, echocardiography and haemodynamic data.

b) SvO_2_ criteria in relation to SAP that could not be explained by shivering, anaemia or hypovolaemia. The criteria were based on extensive studies on SvO_2_ with regard to outcome and clinical experience regarding the approximate relationship between SvO_2_ and SAP while using fast acting vasodilator nitroprusside.^2-5^

SvO_2_ < 50%, SAP < 130 mmHg

SvO_2_ < 55%, SAP < 110 mmHg

SvO_2_ < 60%, SAP < 90 mmHg

SvO_2_ < 65%, SAP < 70 mmHg

**Severe postoperative heart failure**

Patients who fulfilled criteria for PHF were considered to have had severe PHF if it was associated with extended treatment in ICU or death, i.e. criteria 1+2 or 3 were fulfilled.

1) ICU stay ≥ 48 hours (corresponding to at least 72 hours in a unit without a step-down semi-intensive care ward)

2) Use of intra-aortic balloon pump or need for at least one inotropic agent in dosages listed below ≥ 24 hours after admission to ICU

Epinephrine ≥ 0.033 μg•kg^-1^min^-1^

Milrinone ≥ 0.375 μg•kg^-1^min^-1^

Dopamine ≥ 4 μg•kg^-1^min^-1^

Dobutamine ≥ 4 μg•kg^-1^min^-1^

Levosimendan regardless of dose + additional inotropic treatment in dosages above

3) Heart failure leading to mortality

**Preoperative Left ventricular dysfunction**

Moderate left ventricular dysfunction corresponds to an ejection fraction of 0.31 - 0.45 according to echocardiography. Severe left ventricular dysfunction corresponds to an ejection fraction of 0.30 or less.

**Postoperative Myocardial injury**

Myocardial injury was measured with Creatine Kinase-MB isoenzyme (CK-MB) on the first postoperative morning and with Troponin T on the third postoperative day. The time points for sampling were chosen with regard to the different release kinetics of these biomarkers in permanent myocardial injury.^6^

**Hospital mortality**

Hospital mortality was defined as mortality during the first hospitalisation period. This included postoperative stay at the referral hospital as a substantial proportion of patients were discharged to their county hospitals.

**Acute kidney injury**

Acute kidney injury (AKI) was defined as a postoperative increase of p-Creatinine by 50% or more compared with preoperative level in accordance with RIFLE-criteria.

**Postoperative stroke**

Postoperative stroke was defined as neurological or cognitive deficit with a cerebral injury verified on (Computed Tomography) CT-scan. All suspected cases of stroke underwent CT-scan.

**CLINICAL MANAGEMENT**

Clinical management was standardised and similar at the three participating centres with minor differences concerning choice of aneasthetic drugs. After an overnight fast patients received beta-blockers and calcium antagonists orally whereas antihypertensive and antidiabetic agents were withheld. Standard premedication consisted of orally administered flunitrazepam 0,5-1,0 mg or diazepam 5-10 mg and ketobemidone 0,1-0,2 mg kg^-1^ body weight (BW) or morphine 0.1-0.2 mg kg^-1^ BW. Anaesthesia was induced with thiopentone (2-3 mg kg^-1^ BW) or propofol (2 mg kg^-1^ BW) supplemented by a bolus dose of fentanyl 3-5 µg kg^-1^ BW. Muscle relaxation was achieved with pancuronium 0.1 mg kg^-1^ BW or rocuronium 0.6 mg kg^-1^ BW. Anaesthesia was maintained with isoflurane, sevoflurane or propofol supplemented with intermittent doses of fentanyl.

Standard monitoring was used consisting of 5-lead echocardiogram, pulse oximetry, continuous arterial blood pressure monitoring using a cannula in the radial artery, central venous pressure and transoesophageal echocardiography. A surgical pulmonary artery catheter was introduced in all patients.

Standard surgical techniques as described in the manuscript were employed. A median sternotomy was performed in all patients. Twelve patients were operated off pump. In the other 370 patients standard use of CPB and aortic cross-clamping was employed. Single-clamp technique for aortic cross-clamping was used in 54% of the patients. Cold blood cardioplegia was used for myocardial protection in the majority of these patients (78%). At one centre cold crystalloid cardioplegia was used during the first half of the trial. Postoperative sedation was achieved with propofol. Postoperative analgaesia regimen consisted of ketobemidone 7-15 µg kg^-1^ BW administered intermittent intravenously and acetaminophen 1 g every 6th hour.

Extubation was performed when body temperature reached a level above 37°C, haemodynamic values were stable including a mixed venous saturation exceeding 55 %, PO_2_ was above 10 kPa with FiO_2_ 0,4 and PCO_2_ was below 6,5 kPa with a respiratory rate less than 30 and drainage loss was less than 100 ml per hour and declining. After discharge from the ICU patients were transferred to a step-down semi-intensive care unit for at least 24 hours before going to the general ward.

**References**

1. Vidlund M, Hakanson E, Friberg O, et al. GLUTAMICS--a randomized clinical trial on glutamate infusion in 861 patients undergoing surgery for acute coronary syndrome. *J Thorac Cardiovasc Surg* 2012; 144:922-930 e927.

2. Svedjeholm R, Hakanson E, Szabo Z. Routine SvO2 measurement after CABG surgery with a surgically introduced pulmonary artery catheter. *Eur J Cardiothorac Surg* 1999; 16:450-457.

3. Holm J, Hakanson RE, Vanky F, Svedjeholm R. Mixed venous oxygen saturation is a prognostic marker after surgery for aortic stenosis. *Acta Anaesthesiol Scand* 2010; 54:589-595.

4. Holm J, Hakanson E, Vanky F, Svedjeholm R. Mixed venous oxygen saturation predicts short- and long-term outcome after coronary artery bypass grafting surgery: a retrospective cohort analysis. *Br J Anaesth* 2011; 107:344-350.

5. Svedjeholm R, Vidlund M, Vanhanen I, Hakanson E. A metabolic protective strategy could improve long-term survival in patients with LV-dysfunction undergoing CABG. *Scand Cardiovasc J* 2010; 44:45-58.

6. Dahlin LG, Kagedal B, Nylander E, Olin C, Rutberg H, Svedjeholm R. Unspecific elevation of plasma troponin-T and CK-MB after coronary surgery. *Scand Cardiovasc J* 2003; 37:283-287.

**Table S1.** Pre- and postoperative data in patients treated with inotropes depending on if they fulfilled criteria for PHF diagnosis or not

|  | Inotrope use without PHF  (n=55) | Inotrope use and PHF  (n=33) | p-value |
| --- | --- | --- | --- |
| EuroSCORE II | 3.4 [2.0-5.1] | 3.8 [2.6-6.2] | 0.39 |
| NT-proBNP Pre (ng/L) | 660 [426-2430] | 850 [410-1730] | 0.94 |
| NT-proBNP POD1 (ng/L) | 2910 [1833-5165] | 3513[1990-5240] | 0.6 |
| NT-proBNP POD3 (ng/L) | 4290 [2830-6170] | 6850 [3140-12300] | 0.07 |
| Delta NT-proBNP POD3-Pre (ng/L) | 3370 [1658-5060] | 5230 [2850-10290] | 0.044 |
| CK-MB POD1 (ng/L) | 18 [12-23] | 22 [12-35] | 0.041 |
| Troponin T POD3 (ng/L) | 250 [160-530] | 600 [350-1100] | 0.001 |
| ICU stay (hours) | 22 [20-44] | 42 [21-93] | 0.03 |
| ICU stay >72 hours | 13% (7) | 36% (12) | 0.015 |
| Ventilation time (hours) | 5 [3-11] | 8 [6-23] | 0.003 |
| Ventilation time >48 hours | 7% (4) | 21% (7) | 0.09 |
| Postoperative stroke | 5% (3) | 0 | 0.29 |
| AKI | 20% (11) | 36% (12) | 0.13 |
| Hospital Mortality | 4% (2) | 6% (2) | 0.63 |

Data given as medians [interquartile range] or percentages (number).

AKI, acute kidney injury; CK-MB, creatine kinase-MB isoenzyme; ICU, intensive care unit; PHF, postoperative heart failure; POD, postoperative day.

**Table S2**. Multivariable linear regression results for log_10_ NT-proBNP POD1 in all patients adjusted for PHF, glutamate treatment and known preoperative non-cardiac confounders*.

|  | Adjusted coefficient | 95%CI | p |
| --- | --- | --- | --- |
| PHF | 0.164 | 0.064- 0.265 | 0.001 |
| eGFR (mL•min^-1^•1.73m^-2^) | -0.005 | -0.006- -0.003 | <0.0001 |
| Female | 0.176 | 0.098- 0.255 | <0.0001 |

*age, eGFR, female gender, obesity. Adjusted R^2^ = 0.31, ANOVA for the model (df =3, F =46.48,p<0.0001). CI: confidence interval; eGFR: estimated glomerular filtration rate according to MDRD formula; PHF: postoperative heart failure.

**Table S3.** Multivariable linear regression results for log_10_ NT-proBNP POD3 in all patients adjusted for PHF, glutamate treatment and known preoperative non-cardiac confounders*.

|  | Adjusted coefficient | 95%CI | p |
| --- | --- | --- | --- |
| PHF | 0.188 | 0.088-0.289 | <0.0001 |
| Age (years) | 0.011 | 0.007-0.016 | <0.0001 |
| eGFR (mL•min^-1^•1.73m^-2^) | -0.003 | -0.005- -0.002 | <0.0001 |
| Female | 0.104 | 0.029- 0.178 | 0.006 |

*age, eGFR, female gender, obesity. Adjusted R^2^ =0.39, ANOVA for the model (df =4, F =50.26, p<0.0001). CI: confidence interval; eGFR: estimated glomerular filtration rate according to MDRD formula; PHF: postoperative heart failure.

**Table S4.** Variables associated with PHF according to univariable logistic regression

|  | Unadjusted Odds Ratios | 95% CI | p |
| --- | --- | --- | --- |
| Age (years) | 1.12 | 1.01-1.25 | 0.037 |
| Diabetes | 2.16 | 0.93-5.04 | 0.07 |
| COPD | 2.95 | 0.89-9.73 | 0.08 |
| Three-vessel disease | 4.72 | 1.09-20.50 | 0.039 |
| Left main stenosis | 2.21 | 0.98-5.00 | 0.06 |
| AMI < 3 weeks | 3.11 | 1.04-9.31 | 0.043 |
| eGFR ≤ 60 mL•min^-1^•1.73m^-2^ | 1.89 | 0.81-4.38 | 0.14 |
| Moderate LV dysfunction | 2.49 | 0.85-7.30 | 0.10 |
| Severe LV dysfunction | 9.33 | 2.62-33.14 | 0.001 |
| Hemoglobin (g/L) | 0.97 | 0.94-1.00 | 0.049 |
| NT-proBNP Pre ≥ 635 ng/L | 3.22 | 1.40-7.42 | 0.006 |
| NT-proBNP POD1 ≥ 1836 ng/L | 5.72 | 1.67-19.58 | 0.005 |
| NT-proBNP POD3 ≥ 6065 ng/L | 4.46 | 1.94-10.24 | < 0.0001 |
| Delta NT-proBNP POD1 – Pre ≥1372 ng/L | 5.92 | 1.73-20.25 | 0.005 |
| Delta NT-proBNP POD3 – Pre ≥7639 ng/L | 7.30 | 2.91-18.30 | <0.0001 |
| Delta NT-proBNP POD3 – POD1 ≥4299 ng/L | 6.22 | 2.52-15.36 | <0.0001 |
| Aortic cross clamp time (minutes) | 1.02 | 1.00-1.05 | 0.049 |
| Troponin-T POD3 (ng/L) | 1.001 | 1.001-1.002 | <0.0001 |
| Delta Troponin-T POD3-Pre (ng/L) | 1.001 | 1.001-1.002 | <0.0001 |
| p-CK-MB POD1  (µg/L) | 1.019 | 1.007-1.031 | 0.001 |
| Peak postoperative p-Creatinine  (µmol/L) | 1.014 | 1.008-1.021 | <0.0001 |
| Delta p-Creatinine POD3-Pre (µmol/L) | 1.014 | 1.003-1.026 | 0.017 |

AMI < 3 weeks: acute myocardial infarction within 3 weeks of surgery; CI: confidence interval; COPD: chronic obstructive pulmonary disease; CK-MB: creatine kinase-MB isoenzyme; eGFR: estimated glomerular filtration rate according to MDRD formula; LV: left ventricular; Pre: preoperative; POD: postoperative day.

**Table S5.** Multivariable linear regression results for log_10_ NT-proBNP POD1 adjusted for severe PHF*, glutamate treatment and known preoperative non-cardiac confounders**.

|  | Adjusted coefficient | 95%CI | p |
| --- | --- | --- | --- |
| Severe PHF | 0.339 | 0.134-0.543 | 0.001 |
| eGFR (mL•min^-1^•1.73m^-2^) | -0.005 | -0.006- -0.003 | <0.0001 |
| Female | 0.166 | 0.083-0.249 | <0.0001 |

*patients with PHF that were not classified as severe excluded **age, eGFR, female gender, obesity. Adjusted R^2^ =0.29, ANOVA for the model (df =3, F =41.90, p<0.0001). CI: confidence interval; eGFR: estimated glomerular filtration rate according to MDRD formula; PHF: postoperative heart failure.

**Table S6.** Multivariable linear regression results for log_10_ NT-proBNP POD3 adjusted for severe PHF*, glutamate treatment and known preoperative non-cardiac confounders**.

|  | Adjusted coefficient | 95%CI | p |
| --- | --- | --- | --- |
| Severe PHF | 0.258 | 0.042-0.474 | 0.019 |
| Age (years) | 0.012 | 0.007-0.016 | <0.0001 |
| eGFR (mL•min^-1^•1.73m^-2^) | -0.003 | -0.004- -0.002 | <0.0001 |
| Female | 0.103 | 0.026-0.180 | 0.009 |

*patients with PHF that were not classified as severe excluded **age, eGFR, female gender, obesity. Adjusted R^2^ = 0.35, ANOVA for the model (df = 4, F = 41.84, p<0.0001) CI: confidence interval; eGFR: estimated glomerular filtration rate according to MDRD formula; PHF: postoperative heart failure.

**Table S7.** Postoperative data in patients with NT-proBNP POD1<4575ng/L or ≥ 4575ng/L

| Variables | NT-proBNP  <4575ng/L  (n=266) | NT-proBNP  ≥4575ng/L  (n=54) | p-value |
| --- | --- | --- | --- |
| NT-proBNP POD1 (ng/L) | 1789 [1190-2800] | 5500 [4870-9260] | <0.0001 |
| NT-proBNP POD3 (ng/L) | 3035 [1920-4885] | 8700 [5760-15000] | <0.0001 |
| CK-MB POD1 (µg/L) | 14 [10-22] | 20 [12-31] | 0.016 |
| Troponin-T POD3 (ng/L) | 210 [130-390] | 470 [300-800] | <0.0001 |
| ICU stay (hours) | 20 [17-23] | 22 [19-42] | 0.0003 |
| ICU stay >72 hours | 2% (6) | 17% (9) | 0.0001 |
| Ventilation time (hours) | 4 [3-5] | 5 [4-11] | <0.0001 |
| Ventilation time >48 hours | 1% (2) | 13% (7) | 0.0001 |
| Postoperative stroke | 1% (2) | 2% (1) | 0.43 |
| AKI | 9% (23) | 37% (20) | <0.0001 |
| Hospital Mortality | 1% (2) | 6% (3) | 0.036 |

Data given as medians [interquartile range] or percentages (number).

AKI: acute kidney injury; CK-MB: creatine kinase-MB isoenzyme; ICU: intensive care unit; POD: postoperative day.

**Table S8．** Postoperative data in patients with NT-proBNP POD3<6065 ng/L or ≥ 6065ng/L

| Variables | NT-proBNP<6065ng/L  (n=247) | NT-proBNP≥6065 ng/L  (n=78) | p-value |
| --- | --- | --- | --- |
| NT-proBNP POD1 (ng/L) | 1770 [1159-2630] | 4640 [3287-7690] | <0.0001 |
| NT-proBNP POD3 (ng/L) | 2791 [1800-4170] | 8481 [6940-12300] | <0.0001 |
| CK-MB POD1 (µg/L) | 14 [9-21] | 19 [12-29] | 0.002 |
| Troponin-T POD3 (ng/L) | 210[120-370] | 480 [230-760] | <0.0001 |
| ICU stay (hours) | 20 [17-23] | 22 [18-42] | 0.001 |
| ICU stay>72 hours | 3% (7) | 12% (9) | 0.005 |
| Ventilation time (hours) | 4 [3-5] | 5 [3-10] | 0.0003 |
| Ventilation time >48 hours | 1% (3) | 9% (7) | 0.0002 |
| Postoperative stroke | 0.4% (1) | 4% (3) | 0.044 |
| AKI | 8% (20) | 33% (26) | <0.0001 |
| Hospital Mortality | 0 | 5% (4) | 0.003 |

Data given as medians [interquartile range] or percentages (number).

AKI: acute kidney injury; CK-MB: creatine kinase-MB isoenzyme; ICU: intensive care unit; POD: postoperative day.


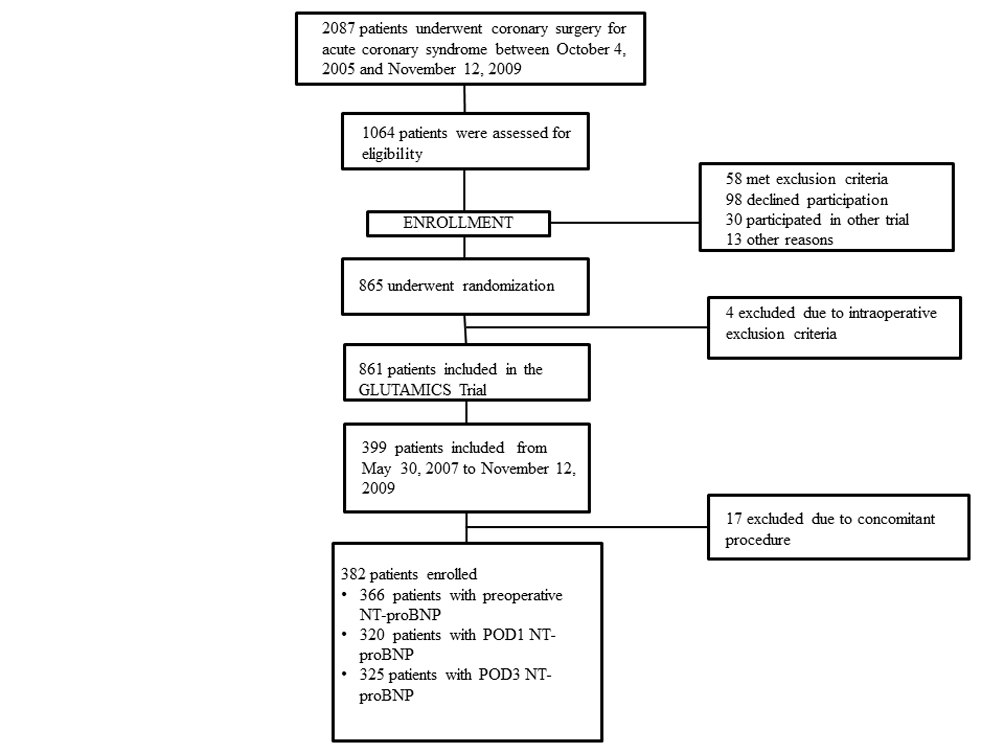


**Figure S1.**  Flow chart of the patients in this substudy of the GLUTAMICS trial. GLUTAMICS trial, GLUTAmate for Metabolic Intervention in Coronary Surgery trial; POD1, postoperative day 1; POD3, postoperative day 3.
